# Explaining how and why social support groups in hospice day services benefit palliative care patients, for whom, and in what circumstances

**DOI:** 10.1177/26323524231214549

**Published:** 2023-12-02

**Authors:** Natasha Bradley, Christopher Dowrick, Mari Lloyd-Williams

**Affiliations:** School of Nursing and Midwifery, Queen’s University Belfast, Medical Biology Centre, 97 Lisburn Road, Belfast, BT7 1NN, UK; Centre for Health & Clinical Research, University of the West of England (UWE), Bristol BS16 1DD, UK; Department of Primary Care and Mental Health, University of Liverpool, Liverpool, UK; Department of Primary Care and Mental Health, University of Liverpool, Liverpool, UK

**Keywords:** emotional support, death acceptance, hospice day care, palliative care, realist evaluation, social support, supportive interventions

## Abstract

**Background:**

Palliative care aims to provide holistic support for people with life-limiting illness, responding to psychological, social and spiritual needs, as well as to clinical and physical. In the United Kingdom, hospice day services (including day care, group interventions, group activities, and social events for palliative care outpatients) aim to provide opportunities for patients to gain social support, which is thought to improve their quality of life.

**Objectives:**

This research explored social support within hospice day services, to explain in detail how and why social support obtained within a hospice day service could be beneficial to palliative care patients.

**Design:**

Qualitative research using observations of hospice day services and interviews with service providers.

**Methods:**

Data collection involved nineteen interviews with hospice service providers (*n* = 19) and researcher observations of hospice day services. The findings detail how patient and hospice context interact to produce mechanisms that lead to outcomes beyond the hospice day service.

**Results:**

Practical, clinical and social aspects of the hospice day service are important for patients feeling welcome and safe in the setting. The opportunity to connect with other people and work towards personal goals can boost self-confidence for patients who have lost access to meaningful activity. New friendships between patients encourages reciprocal support and feelings of belonging. It is beneficial to have permission to speak freely about topics deemed inappropriate elsewhere, because honest communication is helpful in accepting and adapting to their circumstances.

**Conclusion:**

Hospice day services facilitate group settings for reciprocal social support. This research proposes an initial programme theory that can be further developed and tested. It explains how and why, in some contexts, social support increases personal and practical resources to cope with illness and death, leading to changes outside of the hospice (to mood, interpersonal interactions and behaviour) that could improve quality of life.

## Plain language summary

Social support in hospice day services

Hospices provide specialist palliative care to patients with life-limiting illness, offering both clinical and non-clinical support. Some hospices include social settings within their outpatient services which provide palliative care patients with opportunities to meet other people who might have similar experiences (‘in the same boat’). Social support gained within these groups is thought to be beneficial, but there isn’t much research on how and why this might be the case, and in what situations.

We carried out observations of hospice day services and research interviews with hospice service providers. We focused on understanding the need for social support, the social support interactions between group members, and the consequences of these.

The research suggests five processes through which social support within hospice day services could have positive outcomes for patients. These are:

1. Choosing to be there
2. Gaining confidence through new or adapted activity
3. Building familiarity and reciprocity with other patients
4. Honest conversations about illness and mortality
5. Becoming a part of the club

The findings propose how and why, in some situations, social support could lead to improvements in mood, behaviour and relationships. This will be useful to guide the development and evaluation of hospice day services.

## Introduction

Palliative care aims to provide holistic support for people living with life-limiting illness, responding to psychological, social and spiritual as well as clinical needs. Hospices are key providers of palliative care in the United Kingdom. Some offer a range of day and outpatient services that include social support.^1^ Previous research has led a number of authors to conclude social interaction is a core component of palliative day services.^2–5^ Patient and stakeholders are reportedly emphatic on the social benefits of attending – patients appreciate opportunities to meet others, perhaps drawing strength from seeing people cope with similar challenges.^6^ Involving social support in such services aims to reduce unmet social need, alleviate isolation and improve wellbeing;^1^ however, evidence is limited on how and why these social settings can be most effective.^7^

### Literature review

Social support is defined as resources gained through interpersonal interactions and relationships. Social support can fulfil instrumental, informational, emotional or companionship functions.^8^ Inadequate social support may manifest as loneliness or social isolation. Social isolation and loneliness are particularly problematic for people experiencing changes in mobility, limitations in physical function and/or decreasing economic or social resources.^9^ Although the concepts are distinguishable, synergistic interactions between loneliness and social isolation indicate that both are relevant to health and wellbeing.^10^

There is limited evidence on the true prevalence of loneliness in palliative care.^11^ However, a systematic review estimated that 38% of cancer patients (all stages) report moderate loneliness, and the likelihood of loneliness increases in the time since diagnosis.^12^ Furthermore, population-based surveys consistently report people with poor health to be at greater risk of loneliness and/or social isolation. In Australia (*n* = 2222), adults with long-term illness had approximately double the risk of chronic loneliness compared to the general population.^13^ In the United States (*n* = 10,384), people with chronic obstructive pulmonary disease (COPD) requiring oxygen were twice as likely to be socially isolated than those without COPD.^14^ In Canada (*n* = 3613), social isolation became more common as individuals drew closer to death – 18% of people in the last 4 years of life, rising to 27% in the last 3 months – with this association attributable to increasing physical impairment and illness.^15^ Loneliness in this study showed less variation by time to death (18–23% frequent loneliness, 66–72% any loneliness) and was more likely in people with pain, incontinence, or sensory or cognitive impairment.^15^ Finally, in a sample of rural older adults in the United Kingdom (*n* = 884), 49% were isolated from their family and 9% were isolated from their community, people with self-reported physical and/or mental impairment were significantly more affected.^16^

Palliative illness brings additional challenges to obtaining adequate social support, yet coping with life-limiting illness is a largely social process. Maintaining social relationships can be an essential source of meaning and coherence, amidst the difficulties of receiving a terminal diagnosis.^17^ Gaining emotional and informational support from peers can be especially valuable for coping with palliative illness.^18^ Emotional support is commonly reported as a positive coping strategy in patients with incurable cancer and is associated with better quality of life and reduced psychological distress compared to other coping strategies.^19^ Patients seeking social support and positive reappraisal are less likely to experience hopelessness and depression, whereas avoidant coping is associated with increasing anxiety in palliative care.^20^ Social support, especially emotional support, predicts confidence with self-care and symptom management in heart failure.^21^ Perceived social support has also been associated with treatment adherence and quality of life in end-stage kidney disease.^22,23^

Access to and availability of social support influences personal capacity and motivation to aim for better health.^24^ Having a close confiding relationship that reduces feelings of loneliness can be protective against the psychological impact of stressful events and chronic health difficulties.^25,26^ People with many supportive relationships have options to obtain tangible and timely help, including assistance getting to appointments and making decisions about their health.^27^ A social network containing both strong and weak ties is an asset for learning the new skills required for illness management.^28^ Social resources in the present contribute to trust in future support whereas loneliness impacts negatively on self-efficacy and initiative-taking to improve health,^29^ and is associated with increasing frailty over time.^30^

Social networks can be responsive to illness and adapt to increase their ‘illness work’, but this requires resources to be available.^28^ Long-term illness can also restrict social networks: longitudinal parallels are observed in the physical and social decline of patients with malignant and non-malignant disease.^31,32^ People living alone might prioritize autonomy over their willingness to accept help, leaving them vulnerable to low support later in life.^33^ Those who rely on families for support may feel reluctant to burden close relatives with more requests for help while striving to maintain individual independence.^34^ Observable symptoms of illness and fear of stigmatizing responses from other people may also contribute to decreasing social participation.^35^

Patients sometimes keep their worries to themselves due to guilt about burdening other people, shame about the illness, embarrassment of bodily changes or wanting to give the impression of managing independently – leading to a reluctance to disclose that alters relationships inside and outside the family.^36^ Partners, family and friends are unlikely to have first-hand knowledge of what it is like to live with illness and so might be limited in their ability to provide emotional support.^37^ Their own emotional and cognitive reactions to the palliative diagnosis could clash with patient needs – for example, well-intended optimism or positivity that inadvertently dismisses them expressing their concerns.^38^ These interpersonal mismatches (social constraints) thwart opportunities for emotional support, and strain core relationships that are in themselves an important source of identity and belonging.^39^ Social conflict or ambivalence can result in rumination, poorer adherence to health behaviours and other negative coping strategies.^40^

### Research rationale

Unmet needs for social support – including tangible help, emotional support, information and companionship – could worsen the difficulties of living with illness, for both patients and caregivers, and increase the risk of distress towards the end-of-life.^31^ Interventions to provide social support should attend to the ways in which specific contexts shape the needs of their service users^41^; because receipt of social support that is not aligned with the person’s needs or the situation can exacerbate stress.^42^ Interventions aiming to increase social interaction without explored mechanisms of benefit could inadvertently introduce detrimental social experiences, which increase loneliness and/or social withdrawal.^43–45^ However, existing evidence does not provide detail on how and why social support interventions in palliative care could be helpful, and in what circumstances.

### Aim

This research aimed to explain how and why social support interventions might lead to beneficial outcomes in palliative care patients, through qualitative investigation of hospice day services. Informed by realist evaluation, this research develops initial programme theory for social support interventions in palliative care.

## Methods

The study took place as part of a mixed methods PhD project, which included an online survey of social support interventions in hospice day services^1^ and tested the feasibility and sensitivity of patient-reported outcome measures (PROMs) for perceived social support, loneliness and depression.^46^ The qualitative component reported here occurred concurrently to the use of PROMs. Data collection was carried out by NB and involved qualitative interviews with service providers and researcher observations of services.

### Methodology

Realist evaluation is the practical application of the philosophy of realism to the evaluation of complex interventions (also known as ‘programmes’).^47^ Complex interventions provide resources to the people involved, intending to achieve particular outcomes. Yet outcomes are determined by the response of recipients to these resources, which emerges from their context. By asking ‘what works, for whom, in what circumstances?’ realist evaluation contributes ‘programme theory’ to explain how and why an intervention produces change in different situations.^48^

This methodology offers an alternative to study designs that focus on documenting outcomes, by attending to the mechanisms that generate outcomes and the contexts in which these mechanisms occur (occasionally described as ‘the black box’ of interventions). It also contrasts with phenomenological or constructivist methods: by conceptualizing social reality as real, the resulting programme theories may be partially transferable to other situations with similar contexts. Programme theories are developed iteratively, can be tested empirically, and used to inform the development and evaluation of complex interventions.^49^

Realist evaluations embrace complexity, arguing that interventions are not separable from the society to which they are applied.^48^ The goal is to understand causation of intended and unintended outcomes by developing programme theory, usually through the construction of ‘context-mechanism-outcome configurations’ (CMOCs).

CMOCs are theoretical propositions depicting the interconnected causal forces that give rise to outcomes observed when a particular intervention is applied to a particular context. There are always multiple contexts, mechanisms and outcomes, and their distinction may vary between research questions^50^; however, the configuration of these concepts remains a useful tool to interrogate causation. CMOCs assist in developing complexity-tolerant explanations of change, in the form of testable and refinable hypotheses (i.e. programme theories).^47,48^

### Research locations

Nine independent hospices in England participated in qualitative data collection. Research locations were selected to provide variety in hospice size, location and day service aims; and were identified through a survey.^1^ This number of research locations was seen as manageable with the time and resources available; however, diversity of locations was pursued to include rural and urban locations, different regions of England, and levels of deprivation. The hospices involved in this study also provided palliative care on inpatient wards and via homecare, but these aspects of the wider organisation were not investigated.

Research locations were all hospice day services that provided social support primarily for patients in palliative care who usually reside in their own homes (not inpatient at the hospice, or any long-term care setting). These patients would attend the hospice for between 2 and 6 hours, usually on a particular day per week, to participate in group activity (exercise, art) and group discussion (facilitated or unfacilitated) with other palliative care patients. Research locations varied in whether they included transport and a shared meal. Volunteers were present in all day services. Informal caregivers (such as a spouse or parent) were usually permitted to attend alongside the patient.

### Interview participants

The people involved in designing, managing, and delivering an intervention hold implicit understandings of how their programme works – in this case, the intervention is hospice day services.^47,49^ There were between one and four interview participants from each research location, because they varied in the number of relevant services and their staffing levels.

Sampling sought expertise of the day service under investigation, it did not seek diversity of protected characteristics. Several professions were included (nursing, occupational health, physiotherapy, art therapist). The resulting sample of interview participants (*n* = 19) were hospice staff members from nine locations. Participant characteristics are shown in Table 1.

**Table 1.** Interview participants.

Pseudonym	Job title	Time in role or similar	Gender	Ethnicity	Hospice region	Hospice deprivation decile (10 = least deprived)	Urban or rural location
Alice	Support worker	2 years	F	WB	South Coast	5	Urban city and town
Amy	Staff nurse	2 years (20 total)	F	WB	North-West	7	Rural hamlets
Bea	Living well lead	14 years	F	WB	North-West	5	Urban city and town
Carole	Day services leader	4 years	F	WB	South-West	9	Rural village
Danielle	Director of knowledge exchange	8 years	F	WB	North-West	8	Urban city and town
Gemma	Clinical nurse manager	8 years	F	WB	South Coast	5	Urban city and town
Grace	Wellbeing lead	10 years	F	WB	South-East	5	Urban major conurbation
Jill	Creative arts coordinator	15 years	F	WB	South-West	9	Rural village
Jenny	Day therapy manager	9 years	F	WB	South-East	6	Rural hamlets
Katie	Day centre manager	4 years	F	WB	North-West	4	Urban major conurbation
Laura	Sister	19 years	F	WB	North-West	5	Urban city and town
Lisa	Centre manager	10 years	F	WB	North-West	7	Rural hamlets
Lucy	Social care assessor	2 years	F	WB	North-West	5	Urban city and town
Mark	Therapy assistant	6 years	M	BB	South-East	5	Urban major conurbation
Mary	Physiotherapist	10 years	F	BB	South-East	5	Urban major conurbation
Patricia	Day hospice manager	6 years	F	WB	South-West	10	Rural town and fringe
Rachel	Head of rehabilitation & wellbeing	8 years	F	WB	South-East	5	Urban major conurbation
Sarah	Art therapist	2 years (20 total)	F	WB	South Coast	5	Urban city and town
Shauna	Healthcare assistant	18 years	F	WB	North-West	5	Urban city and town

F, Female; M, Male; WB, White British; BB, Black British.

Inclusion criteria were for hospice personnel to be currently responsible for the design, delivery or management of a hospice day or outpatient service that included a component of in-person and indoors social support for palliative care patients. Interventions taking place outside of the hospice building, in an outdoors setting, or within the patient’s home were excluded as out of scope for this investigation.

### Recruitment

Interview participants were invited to participate by a manager or service lead and provided with brief written materials and a participant information sheet. The service manager or lead identified relevant and available staff and introduced the researcher to them. All participants who were approached agreed to be interviewed. Participants had the opportunity to ask questions before deciding whether to take part. Written consent was obtained prior to the interview.

### Interview procedure

Interviews took place in a private room at each hospice. They were audio-recorded and transcribed. A topic guide was used, but the interview allowed for new or unexpected topics to come up. Specific questions from the observations were brought into the interview, and the researcher invited the participant to give feedback on their early reflections about context and causation.^49^

### Researcher observations

Observations of hospice day services by the researcher were conducted as focused ethnography. Ethnographic methods allow researchers to observe situated, everyday social interactions while immersed in the field of study. In this study, observations were conducted as a focused ethnography, characterized by shorter lengths of time often in settings that are part-time rather than operating continuously.^40^ The researcher observed the social settings within hospice day services at six locations for between 1 and 4 days (11 full days in total between August 2018 and April 2019). Staff and volunteers were provided with brief written materials introducing the project before the researcher’s arrival, and consent was given verbally from patients present prior to the researcher observations of services.

Observations allow the researcher ‘to see directly what someone does, rather than what they say’ – they contribute different types of information and can help to broaden the types of people included in the project.^51^ Observations allowed for informal conversation with patients and volunteers, who could spontaneously offer comments on the service to the researcher, as and when they chose to do. This approach was seen as preferable to a formal interview, and more acceptable to hospice gatekeepers at the time, because it was less disruptive to the patient’s day and avoided risk of inadvertently upsetting patients experiencing loneliness or isolation. The observations served to inform and deepen the interviews and analysis, because the researcher was knowledgeable of practice and had directly experienced aspects of the intervention and the interaction within.^49,52^

During observations, the focus was on interpersonal interactions between patients, because the quality and content of these interactions constituted accrual of social support. Information on sensory input and pace of the services was also captured as relevant to the social experience of the setting. To reduce conspicuousness, the researcher participated in the activity and fieldnotes were primarily made immediately afterwards, with only brief comments or short quotes jotted down during the day as memory prompts. Journal entries in the morning and evening were intended to reveal and bracket pre-conceived expectations and to document the researcher’s emotional state before and after each observation.

Three hospices were not able to participate in researcher observations due to changing or pausing their day service delivery at the time. Instead, the researcher had a tour of the building prior to the qualitative interview.

### Data analysis

Written transcripts from interviews and fieldnotes were imported into NVivo (Lumivero). Analysis was conducted by NB and discussed with MLW and CD. The first stage of analysis coded for hospice resources; mechanisms occurring intrapersonally or interpersonally within the hospice; processes occurring beyond the hospice walls; reported contextual influences and outcomes for different stakeholders.^53^ Thematic summaries of each of these areas (resources, contexts, mechanisms, outcomes) were presented to the research team so that the consistency of coding could be assessed. Subsequent theory development considered how the connections within and across different social levels (i.e. personal, interpersonal, institutional, societal) indicated salient interactions between aspects of the patient and hospice context which contributed to the generation of mechanisms, and the outcomes considered to occur as a result.^48^

This process allowed for the drafting of early CMOCs, which were discussed within the research team and then refined by moving iteratively between transcripts, fieldnotes and codes. Finally, CMOCs were considered for plausibility by referring to relevant substantive theories^54^ of social comparison theory, self-determination theory and existential psychotherapy.^55–57^ The CMOCs are intended to provide a parsimonious account of how and why outcomes are generated within a particular context.

In the following section, five CMOCs are presented, each derived from this iterative analysis using interviews and observation data. Each CMOC is preceded by a narrative explanation including illustrative quotes from the interview participants.

## Findings

### Choosing to be there

The need for social support interventions arises due to the multiple ways in which living with life-limiting illness impacts on the social experience of patients and their caregivers. Daily activities might be restricted by symptoms including fatigue, pain, breathlessness or loss of mobility. Managing healthcare appointments and care arrangements, and the side effects of treatment are further drains on time and energy. Taken together, these aspects make it more difficult to spend time with other people by substantially increasing the ‘work’ required to make and execute social plans. Low energy, low mood and loneliness can reinforce each other over time. Interviews thus suggested a path from illness symptoms to withdrawal and isolation, such that some friendship groups might break up or no longer include the patient.

Gemma: Some of them feel a total burden, some of them get quite agitated and frustrated with what they can’t do – ‘I can’t do the gardening anymore, I can’t put that lightbulb on, I’ll have to ask my wife to do it’. – There’s a lot of frustration with just sitting at home, a lot of reminding of the losses that they have to go through.

Receiving a palliative diagnosis can bring feelings of uncertainty, fear and powerlessness. Shock about the diagnosis and worries about the future might correspond to unmet needs for emotional and informational support that become motivating factors in them choosing to attend the hospice day service. People vary in their reasoning for choosing to come – they might be sociable people by nature, be specifically motivated to connect with other patients, or attracted to other intervention components. They might be keen to spend time in a new place, or hoping to give their spouse or caregiver a break.

Jenny: The people who are struggling more with their diagnosis, struggling more with the changes in their physical self, their emotional self, they need to talk to people who understand what they’re going through.

Interviewees explained that everyone should feel welcomed and included in the space, but also able to engage at their own pace and in their own way. Freedom to move between rooms and to make independent decisions on goals was frequently emphasized. Making positive changes requires conviction that positive change could be possible in our circumstances, but patients might feel lacking in reasons to hope in their existing context. Making the choice to attend the group could feel as a positive step towards trying something new, though the atmosphere for the first visit would be important in confirming this perception. A common idea across interviews was that attendance at the hospice day service provided something to look forward to.

Patricia: A lot of what we see is people who have lost meaning in their life. They haven’t just been a sick person, they’ve done a myriad of wonderful interesting things, and suddenly they’re being cared for and they’re being done for and they are a selection of other people’s tasks and that’s not very meaningful. There aren’t just those people that just want to hang out, just like your company. . . Day hospice is really a choice to add extra to life.

#### Context-mechanism-outcome  configuration

A person’s normal routine and access to social support is disrupted by declining or fluctuating health, and the social and emotional challenges of life-limiting illness. Unmet needs motivate some people to seek or accept help – such as deciding to attend a hospice day service. This requires their attendance to be both practically feasible and socially acceptable – they have knowledge of the hospice that determines their expectations of potential benefit and thus willingness to attend (Context).

The hospice day service offers a variety of options for engaging, flexibility between or within day services and an emphasis on personal goals. Patients have a sense that they have made a choice about being at the hospice day service and what they do there, which encourages a sense of autonomy and personal control in the experience (Mechanism).

The room is filled with people who have chosen to be there – and this adds value to the experience of being there. Patients feel hopeful that they could derive benefit from attending (Outcome).

### Gaining confidence through (new or adapted) activity

Life-limiting illness can force people to step back from ‘pre-illness’ social activity – including their chosen occupation, sports and hobbies, or even their position in the family. Losing access to these sources of purpose could bring sadness at not being able to live the life they had hoped for. Maintaining personal care and domestic tasks becomes more difficult which could further impact confidence. Expectations of illness within the family might be directly unhelpful, if relatives are limiting opportunities to participate in activities by discouraging any exertion. Patients can also have anxiety about symptoms becoming aggravated whilst out and about, and how this would be received by other people.

Enjoying the atmosphere and finding new activities at the hospice day service might then be a stark contrast to other experiences arising since the illness, especially for those who have barriers to leaving the home. Activity within the space offers a break and challenges assumptions about the passivity of illness. Having a goal is useful in and of itself: something new to aim for, and a break from rumination on clinical progress. Examples could be getting into a car, being able to play with grandchildren, or better stability walking with friends.

Mark: Maybe ‘cause we’re not doctors, you know, we’re not based on a ward and it’s something different, gives people maybe a different focus as opposed to focusing on their illness, they can just focus on the task in hand. And so, whether it’s coming to the gym, or doing something and taking ownership of it, just aiming for something, it might give them a different way of thinking. Once people know what they can do and what they can be capable of, that gives them a boost.

Activity is also an effective way of getting people together: facilitated conversations around a shared activity are a key component of hospice day services. These conversations might be most useful because topics are free flowing; and the sensory distraction enables deeper communication to take place (whether through art or through exercise). The chat can become meaningful emotional support or useful information exchange, without having to be guided by a professional agenda.

Katie: Her sense of worth just increased and she started doing more, she was laughing again. . .I saw her husband and he just said I feel like I’ve got my wife back.

Within the social setting, attendees are more able to fulfil previous ‘pre-illness’ social roles – class clown, host, big sister – and talk about their interests in a relaxed environment. The group setting might enable the patient to reapproach activities they had previously enjoyed, such as gardening or carpentry, by providing advice and encouragement for appropriate adjustments. For patients with a high level of health anxiety, their perception of clinical safety could be a prerequisite for other positive experiences in the space. If the day service does improve their confidence, then patients could become more able to try new things and make positive changes outside of the hospice.

Lisa: They know it’s ok to try something here, because there’s that back-up of the nurses here or maybe equipment needed to manage a situation. If they can try something here, when they go home, they might do that again, and not feel so worried about it because they’ve got more confidence.

#### Context-mechanism-outcome configuration

Changes to function or appearance can leave palliative care patients less able to participate in their meaningful activities, and they may be treated differently by other people in their lives. These experiences erode confidence, such that declines in physical function might be accompanied by increasing social restriction and feelings of emptiness (Context).

Through identifying and working towards a goal within the hospice day service, patients might gain personal confidence. Despite all their limitations, patients may be learning new things and witnessing other people’s achievements. These experiences are enabling, in that they build hope for positive change (Mechanism).

Mood lift makes a difference to functional ability and to participation in daily activity, and these benefits reinforce each other over time. This means that patients could experience increases in confidence and physical ability they did not expect; or go on to do other things that might not have seemed accessible to them beforehand (such as coffeeshops or local gyms) (Outcome).

### Building familiarity and reciprocity

The illness and its consequences might disrupt previous social routines and opportunities to contribute. A driver towards social withdrawal can also be the reactions of friends and family members to the diagnosis and realities of illness. Patients can struggle to cope with visible changes to their appearance – signs that the body is functioning in a different way to ‘normal’ can be a stigmatizing reminder that the person is ‘sick’. If they blame themselves for their illness, there might be feelings of guilt that increase self-consciousness around body image. Some patients feel unrecognizable and dodge those that remind them of their changes since the illness. They might feel a sense of alienation from others if close friends seem unable to relate to them since the illness.

In contrast, meeting people who do not know the ‘pre-illness’ self can be helpful, because a new person is less likely to express shock or sympathy at the change. Feeling at equal with each other is a foundation for trusted exchange: shared experiences between patients are a source of useful advice (informational support) and empathy (emotional support). There can be a sense of normality in being in a room of patients, in that conversation can move past the illness and towards the person – questions like ‘how are you today’ aren’t an interrogation into symptoms or prognosis, but genuine queries into how you are feeling as a person.

Danielle: Maybe if you’re ill and somebody else is not, that makes you different, maybe it makes you feel weaker. Maybe if you’re talking to people that are ill like you are, the illness becomes smaller, so you talk about other things. If you’re mixing with everybody that’s in a similar situation, you are talking about the football, because you’re not different, you’re on a more level playing field and there’s a bit of mutual help there, you can give as much as you can receive.

Knowledge received from a professional can feel less relevant than first-hand experience. This means that the camaraderie between patients can provide the needed encouragement to make changes to adapt to illness. The group environment could help overcome the stigma of accepting equipment, such as a walking stick, because familiar peers can emphasize its practical utility rather than its social connotations.

Carole: It enables some of the patients to give, and I think that’s important as a human being, to have something to give. You might’ve lost your ability to do the washing up and mow the lawn and all those kinds of things, but actually, if you’ve felt you’ve given something to somebody, even words of comfort, or you know you’ve distracted them for half an hour, or whatever – they get a lot from being able to give to each other. I think that’s really important. It’s one of the few things some of them can still do.

Familiarity over time helps to normalize experiences and leads to deeper communication. The process of giving support to others seems crucial – especially if the patient’s context means other helping roles have been taken away through their illness. The hospice day service provides valuable opportunities to give useful advice, emotional support, a joke or just some company and genuinely help someone else in the process.

The emotional side of palliative care can be overlapping between diagnoses, meaning that authenticity is probably more essential than exact clinical similarity. Furthermore, it is the differences between patients, in their diagnosis and prognosis, that helps them appraise their own condition, experience empathy for each other, and perhaps shift their reasoning. When they are the only person they know living with illness, there are fewer opportunities for the patient to reflect on the relative upsides of their own situation.

Jenny: It’s amazing how often people will say I thought I was bad until I saw them, and that actually gives people quite a lot of strength. Last week I overheard one of the guys saying to another guy ‘You’re my hero. When I see you and what you cope with, it makes me realise I really haven’t got anything to complain about’. Seeing other people who are ill, but not necessarily having the same illness as you, I think is valuable.

#### Context-mechanism-outcome configuration

Life-limiting illness can make socializing practically more difficult. This could be particularly so where there are stark changes in appearance, feelings of guilt about lifestyle choice or the desire to protect other people from negative emotions. Emotional loneliness and loss of social contact can feel especially painful when coping with a short prognosis (Context).

Being in a room with fellow patients feels different to other social settings. Getting to know other patients changes the social resources available, and this helps to reframe or reappraise their situation (Mechanism).

Meeting other patients demonstrates they are not coping alone with the illness. Patients learn from each other, and this can develop into reciprocal friendships which are a lasting source of support (giving and receiving) after discharge – support that is not based on sympathy or obligation, but rather a willingness to listen and understand during the bad days (Outcome).

### Honesty (and humour) on illness and mortality

Receiving a palliative prognosis can bring emotional turmoil and fear; and there may be increased thoughts of death, decline and their own dying. Alongside the diagnosis itself, patients may be processing other distressing experiences within the healthcare system, such as sudden or extended hospitalization. Additionally, patients and caregivers are tasked with navigating the responsibilities of illness management, including appointments and care arrangements in the present, and difficult decisions in the future. Some might feel a sense of abandonment if they feel they’ve been left to manage for themselves at the end of curative treatment.

Close relations who are confronted with illness and symptoms that can’t be easily fixed or managed might also struggle to handle the prognosis. The shock of a palliative diagnosis amidst family dynamics of stoicism or protection can contribute to patients feeling unable to talk about how they’re feeling, wanting to protect their loved ones. Wishing to talk about death might be seen as ‘giving up’, whereas patients might want to talk about the changes they’re experiencing – to their body, daily life or future wishes.

Bea: We hear that an awful lot – I can’t talk about death and dying to my family because I’ll upset them, or they don’t want to talk about it, they think I’m morbid.

It’s really important because if you have been given a life-limiting diagnosis, the immediate thing you do is to jump to death, you cut out the middle bit, you start planning your own funeral. . .. Patients are the ones that have been through it, and we haven’t been through it, so we don’t know what it’s like. But we’re giving them permission to say what it’s like and listening to that and valuing that.

Time spent in isolation can contribute to rumination and anxiety about health. People who had previously been content living alone could need more support than is available to process the diagnosis and make decisions about care. Help-seeking behaviour that is motivated by fear or anxiety is more common for patients who have unanswered questions and cannot easily gain reassurance easily at home. Some patients have abundant friends and family from whom to gain guidance – for others, the only trusted source is via healthcare services.

Lucy: They don’t want to bother their families, because they are already doing enough for them, taking them to appointments and so on.. . . I can only imagine it must be lonely, because you’re trying to put a brave face on in front of your loved ones.

In contrast, social opportunities characterized by choice and activity could facilitate emotional disclosure and communication that supports patients in processing the change and losses they have already experienced and expect to experience. Advance care planning can be daunting, but the discussions within the hospice environment help plans to be put in place and care to be coordinated. Patients might be able to be more honest about their thoughts because the space is separate from home life. During their time spent in these settings, patients can have practice conversations, or share on ‘inappropriate’ topics, without upsetting their family members. Humour is an important process for communication and personal acceptance. Within the group, they can laugh at things they might not be able to express at home. By admitting a change to someone else (even with a joke), they begin to admit it more deeply to themselves.

Gemma: They can share and be open about stuff. The chaps can get together and talk about wives and the sexual dysfunction bits and bobs, stuff they perhaps thought they would never be able to share with anyone. . . It’s kind of safe because they don’t really know each other

#### Context-mechanism-outcome configuration

Distress can arise from actual or anticipated changes in the self – it is difficult to process the knowledge that the physical self has changed, is changing, and will continue to change. But patients might feel unable to express how they’re feeling, wanting to protect their loved ones from having to talk about death or acknowledge the loss. This can leave them feeling alone in the decisions they are facing (Context).

The hospice day service provides opportunities to talk with peers about illness and mortality. Sharing within the hospice day service can even feel an altruistic act that could help someone else in the group. Because the space is separate from home life, patients can rehearse conversations, with less concern for impact on other people (Mechanism).

Conversations within the day service could help to deconstruct death avoidance, and thus enable consideration of one’s mortality, which is necessary to identify preferences and opportunities for agency towards the end of life. Patients may feel less fearful, or have more peace of mind, having shared their fears around sickness and death. They may have benefitted from processing emotions around mortality within the group, allowing for mood improvements in daily life (Outcome).

### Becoming a part of the club

The hospice provides patients with a space to connect with other patients, obtaining comradery and companionship through group activities. As equals, patients can give reciprocal support and build trust in each other. They might feel this is a group that accepts them and their illness-related changes, while allowing them to be who they are beneath the illness. They are becoming ‘a part of’ the club, in contrast to ‘apart from’ other people. This can give rise to feelings of belonging that are particularly potent for people who are experiencing alienation or powerlessness in other situations.

Carole: One of the big challenges of getting sick is the roles that you used to have are not there anymore. You either haven’t got the energy or you physically can’t do it anymore. You are lost, you aren’t that person that you always thought you were. It’s really difficult – without an identity you feel worthless, you feel useless, it’s really challenging.

A new sense of purpose could arise from aiming to contribute to the hospice group. Awareness of the time and devotion from staff and volunteers might contribute to a sense of trust in the support available, and credibility of the hospice as a community that will be there for them in the future. Activities that enable altruism towards others, including patients beyond the day service or towards hospice fundraising activities, could provide additional opportunities to enrich feelings of belonging to a community.

Rachel: We want to be much more about a community or a place or a support network that is ongoing, but it isn’t always all about the professional specialities, there’s this connection with volunteers and with other people who are also living with the same conditions or problems, so that we connect people but we’re not the sole caregivers, the ones that people come to rely on.

Meeting other patients can demonstrate that positive change is possible. However, those attending a hospice day service might also experience death of group members. This ‘reality check’ motivates future planning – a challenging confrontation, that allows the person to consider their own preferences, fears and opportunities for agency towards the end of life. In some cases, for patients to witness each other’s journeys gives them reason to have hope about their own.

There is an additional importance to properly acknowledge the death of group members. Reflecting on the passing of former group members might allow patients to reflect on how they too will be remembered. A small sense of legacy within the group is especially valuable for those who are socially isolated, who may not feel their death will have an impact in other settings. Staff are not immune to the loss; by processing the death together, the group perceives how each death is marked with respect.

Gemma: One person said this has really reassured me because when I die I know I’m going to be remembered and I think we were all quite emotionally affected by that comment, but its real reassurance . . . it’s really important to remember people, for both, for the patient and for the group

#### Context-mechanism-outcome configuration

Loss of normal life due to the illness for some patients can lead to a cycle of low mood and social isolation. Functional losses and emotional estrangement from friends could fuel withdrawal from social participation (Context).

The hospice resources facilitate ‘holistic safety’ – physically, clinically, socially safe – in which the group setting can facilitate patients in moving towards personal goals, new friendships and enriched communication. Their awareness of time and devotion from staff, volunteers and peers might lead to a sense of trust in the support available, and credibility in the hospice as a community of people (Mechanism).

Feelings of belonging and purpose are psychologically potent experiences. Ideally, group members feel the group accepts them and allows them opportunities to be who they are ‘beneath the illness’. The connection to the community allows them to move towards acceptance of physical and existential limitations (Outcome).

## Discussion

### Summary of findings

Hospice day services are multicomponent and multidisciplinary interventions. This research foregrounds social support as a vital part of what patients can gain from day services. The findings outline five processes through which the psychosocial wellbeing of the patient could be improved through the interpersonal and group interactions within the intervention:

1. Choosing to be there
2. Gaining confidence through activity
3. Building familiarity and reciprocity
4. Honesty (and humour) on illness and mortality
5. Being a part of the club

These would vary in importance between individuals and over time. Together, they explain how and why hospice day services provide social resources that might lead to beneficial outcomes. To summarize, social support within hospice day services could contribute to patient motivation and ability to participate in activities of independent living, which include regular and meaningful social contact (to ‘live well’); they could also contribute to them being able to talk about, plan and achieve, their end-of-life wishes (to ‘die well’). These outcomes lead to improved health (understood holistically) that enable better living and dying experiences for patients and their caregivers, and could have positive ripple effects for families, healthcare professionals and the wider healthcare system.

### Discussion with literature

This section briefly outlines supporting literature for the significance of these processes, thus detailing how the overarching concept of social support can have positive impact for people in palliative care.

#### Choosing to be there

Factors contributing to patient loneliness include personal inauthenticity (‘a brave face’), social constraints, traumatic healthcare experiences and the societal stigma of illness and dying.^58^ The day care centre is an area or space that is supportive and responsive to its clients.^4^ Attending and eventually belonging to a new social group has the effect of changing the social surroundings of the patient, if only for a few hours a week at first. Being in a bound space can have consequences for feeling safe to share: within this space, they have permission and support to approach topics or activities that have previously been denied.^56^

#### Gaining confidence through activity

Patient loneliness and fears of becoming a burden may stem from a context of present and future threats to personal autonomy.^31^ Activity and safety within the hospice day service can help to boost mood and regain confidence, helping patients to rediscover meaningful activities. This suggests that social support interventions go beyond relational changes when helping palliative care patients preserve a sense of purpose, allowing threats to be redefined with new meaning.^59^

#### Building familiarity and reciprocity

Addressing social support within a group intervention for advanced illness can lead to mental health benefits.^60,61^ Coping together allows for ‘invisible’ support, whereas one person obviously helping another is unhelpfully ‘visible’.^62^ Passing on help to other people might restore self-confidence after receiving help yourself,^63^ and self-compassion can be stirred by showing compassion towards others.^64^ Opportunities to connect with other patients as equals helps to alleviate emotional loneliness; but in order to benefit fully, patients and carers might need the time and opportunity to build trust and a sense of community.^3^

#### Honesty on illness and mortality

People who are constrained in talking about their illness (or other trauma) are less able to process their experiences – thus the psychological benefits of social coping are moderated by the responses of others.^65^ Previous detrimental interpersonal interactions can create reluctance to disclose, even when support is available.^48^ Interpersonal interactions occurring over time, from a basis of shared experience, lead to great respect between group members. Patients might feel they can be honest with each other, and a sense of humour can develop that might not feel possible in other settings.^66^

#### Being a part of the club

Through shared experiences and honest communication, each group member becomes a living inspiration to the others: this phenomenon goes beyond dyadic empathy and is paramount for hope. The great strengths of support groups such as ‘Alcoholics Anonymous’ has been attributed to the conviction developed by group members that ‘they can be understood only by someone who has trod the same path as they’.^67^

This analysis emphasizes how management of existential anxiety could be enabled through social support, especially through behaviours that are altruistic or otherwise identity-reinforcing (role enacting) or legacy-making. Performing (and remembering) these interactions could be protective against psychological distress in the context of mortality salience. There is value in the group bond, but also in the separation of members, in the meeting of people from entirely different life courses and social networks. Members learn what they can and cannot obtain from others – including escape from death.^67^

### Limitations

This research explored how and why social support interventions provided by hospice day services may be beneficial, by observing the services provided and interrogating the perspective of service providers. A limitation of this work is that the patient voice was not formally sought out during the investigation. However, patients, caregivers and volunteers had opportunities to informally speak to the researcher during the observations – often expressing their enthusiasm about the value of attending these group settings. Data was collected prior to the COVID-19 pandemic, which did disrupt service provision and may have had a lasting impact on the social support needs of people living with life-limiting illness. Future research should therefore consider the patient’s perspective and how their experience of social support interventions might be influenced by changes in the wider context.

Our own interest as a research team in social support led us to carrying out this research, and it is possible that participating research locations were more interested in social support than non-participating locations. Interview participants included a range of ages and professional backgrounds and were usually residents of the local area served by the hospice. We did not seek out interview participants who disagreed with the provision of social support interventions, which could be a further limitation of this work.

### Implications

Living with life-limiting illness impacts on the social experience of patients and informal caregivers, which gives rise to the need for social support interventions. Non-clinical aspects of palliative care are important to patient and caregiver experience and help to achieve the intended outcomes of more costly interventions. Hospice day services can be impactful for wellbeing by providing palliative care patients with opportunities for group activity and unstructured conversations that give rise to new friendships, altruism, reminiscence and humour. Time spent together is relevant to the development of friendships and might be reduced in some appointment-based models.

The need for social support intervention could be more pronounced in rural or deprived areas, where there are additional limitations on access to social support. However, reaching the most socially isolated people remains challenging. Welcoming a range of patients requires hospice personnel with a breadth of skills and knowledge – this requirement could become more salient when increasing the scope of diagnoses, ages and cultural backgrounds served by the hospice. Some people may find same-gender groups to be helpful to have permission to talk honestly about ‘inappropriate’ topics.

Effective social support could lead to positive and lasting change outside of the hospice. However, ‘taking it home’ is not guaranteed – it might be limited by aspects of the patient’s context such as family attitudes, financial resources, and the physical environment (restricted mobility or accessibility). There was a broad variation in the functional disability of people attending the groups included in this study. It is possible that shifting emphases on empowerment and autonomy with hospice day services will impact on the inclusive potential of social support derived in these settings. Sensitivity is needed to unpick the interaction of empowerment narratives from the accessibility of these services for the most disabled patients.

## Conclusion

Palliative care patients can experience loneliness and social isolation from the losses associated with illness, their changing support needs, and constraints on emotional communication. Opportunities to help or give back by sharing information, emotional support and companionship might be a crucial ingredient in alleviating patient loneliness, particularly when thoughts of dependency and decline are fuelling distress.

The initial programme theory developed in this study explains how social support within the hospice day service could address unmet needs and increase psychosocial resources to cope with illness and prepare for dying. Further work is necessary to explore patient and caregiver perspectives and to establish appropriate outcome measurement for intervention study.

## Declarations

**Ethics approval and consent to participate:** Research was conducted in accordance with the Declaration of Helsinki. Ethics approval was provided by the University of Liverpool Faculty Research Ethics Committee on 07/02/2017 (Ref: 1266). Informed consent was obtained from interview participants *via* a written consent form. Informed consent was given verbally prior to researcher observations of services.

**Consent for publication:** Participants gave consent for anonymized quotes to be published.

**Availability of data and materials:** Full data sets will not be made publicly available due to sensitive nature of transcripts that might compromise the anonymity of participants or reveal patient information.
